# Can Zoos Ever Be Big Enough for Large Wild Animals? A Review Using an Expert Panel Assessment of the Psychological Priorities of the Amur Tiger (*Panthera tigris altaica*) as a Model Species

**DOI:** 10.3390/ani10091536

**Published:** 2020-08-31

**Authors:** Jake Stuart Veasey

**Affiliations:** School of Animal, Rural and Environmental Sciences, Nottingham Trent University, Southwell NG25 0QF, UK; jake@carefortherare.com

**Keywords:** Amur tiger, animal welfare, zoo, psychological priority, behavioural need

## Abstract

**Simple Summary:**

The reduction in space available to wild animals in zoos and aquariums is widely perceived to be detrimental to their welfare by scientists and the general public alike. Evidence suggests that naturally wide-ranging carnivores are more likely to suffer in captivity than those that travel less widely. Using the Amur tiger as a representative for wide-ranging species frequently held in zoos, an expert panel assessment was undertaken to identify psychological priorities in order to see how the negative welfare impacts of reduced ranging opportunities might be most effectively overcome. This assessment highlights that whilst reduced access to space may be central to compromised welfare for many species, there may be more effective strategies in safeguarding welfare than simply making captive habitats marginally bigger. Central to this for Amur tigers is providing appropriate mental stimulation rather than focusing only on behaviours linked to hunting. Various strategies intended to safeguard welfare are discussed for Amur tigers, which can also be considered for other wide-ranging species.

**Abstract:**

The ecology of large, wide-ranging carnivores appears to make them vulnerable to conservation challenges in the wild and welfare challenges in captivity. This poses an ethical dilemma for the zoo community and supports the case that there is a need to reconsider prevailing management paradigms for these species in captivity. Whilst the welfare challenges wide ranging carnivores face have been attributed to reduced ranging opportunities associated with the decreased size of captive habitats, attempts to augment wild carnivore welfare in captivity typically focus on behaviours linked to hunting. Thus far, this has yet to result in the systematic elimination of signs of compromised welfare amongst captive carnivores. Here an assessment is carried out to identify the likely welfare priorities for Amur tigers, which, as one of the widest ranging terrestrial carnivores, serves as an excellent exemplar for species experiencing extreme compression of their ranging opportunities in captivity. These priorities are then used to consider novel strategies to address the welfare challenges associated with existing management paradigms, and in particular, attempt to overcome the issue of restricted space. The insights generated here have wider implications for other species experiencing substantive habitat compression in captivity. It is proposed here that the impact of habitat compression on captive carnivore welfare may not be a consequence of the reduction in habitat size per se, but rather the reduction in cognitive opportunities that likely covary with size, and that this should inform strategies to augment welfare.

## 1. Introduction

The welfare of wild animals in captivity is rightly an area of growing public awareness, and the size and naturalness of captive habitats are perhaps the most widely perceived causes for concern amongst the public [1,2]. Many advocates for animal welfare, including welfare scientists, argue that the limited scale and complexity of captive environments, and/or a lack of opportunity to be truly ‘wild’, result in widespread compromises to wild animal welfare [3,4,5,6,7,8]. However, before considering whether or not specific captive states compromise welfare and what strategies might be required to improve welfare for a specific species, for the purpose of this assessment and review, it is essential to clarify what is meant by animal welfare as it is a construct with many nuances and diverse interpretations which are prone to changes over time [9,10,11,12,13].

Here, animal welfare is considered in relation to the affective or emotional states of animals (see [13,14,15,16,17,18,19,20,21,22,23]), with poor welfare occurring when an animal experiences severe or chronic states of mental suffering and good animal welfare occurring when an animal experiences positive emotional states and negligible mental suffering [20,23]. However, because animals cannot communicate their emotional states directly with us, feelings such as happiness and unhappiness remain impossible to measure directly [24,25] and difficult to assess indirectly (see [9,13,18,22,26,27,28]). Veasey argues that the challenges associated with assessing the feelings of animals has resulted in animal welfare assessment, and subsequently animal welfare management [13], focusing unduly on metrics linked to physical health [13,15,19] at the expense of the psychological wellbeing of animals, and that this reflects an inherent tension between physical and psychological priorities in captive animal welfare management [13].

According to Veasey, this tension routinely negatively impacts the wellbeing of wild animals in captivity, including that of large wide-ranging carnivores [13], and illustrates this point by referencing guidelines produced by the American Association of Zoo Veterinarians (AAZV) which state: “When possible the use of species specific, commercially prepared animal diets should be utilized as the basis for any nutrition program.” [29]. The approach advocated by the AAZV has been widely adopted within the zoo sector but perhaps most especially within North America, where, for example, the provisioning of daily processed meals to many large felids is widespread. Veasey argues that whilst such meals are sufficient to maintain physical health, they are not optimised for the psychological needs of animals [13]. Feeding large felids small meals on a daily basis as opposed to less frequent, larger carcass-based feeds typical of the group’s diet in the wild [30] eliminates naturally occurring, powerfully motivated behaviours and prevents sufficient stomach distension which would naturally suppress the motivation to forage [13,31]. Thus, captive large felids fed smaller processed meals will likely be chronically motivated to forage, whilst simultaneously being frustrated at being unable to do so [13]. Whilst the provision of carcass feeds for carnivores might facilitate the expression of important species-specific behaviours and provide opportunities to experience positive affective states [32,33], including satiety and with it the natural suppression of the motivation to forage [13,31], it also exposes animals to a most likely modest increase in the risk of choking and digestive complications related to bone ingestion, or nutritional deficiencies related to the lack of control on portion size and quality, which processed diets can eliminate. When faced with the hypothetical but nonetheless tangible risk of death or complications linked to carcass feeding versus its potential and imprecise benefits, which are notoriously difficult to quantify (see [10,13,18,22,26,27,28]), it is perhaps understandable why those responsible for determining management strategies might focus on the physical components of nutrition at the expense of the psychological components and opt for processed diets as recommended by the AAZV [29].

As this example illustrates, in order to optimise captive animal welfare, it is necessary to balance the benefits captivity affords in terms of protecting the physical wellbeing of animals with the need to provide animals with opportunities to express those behaviours and cognitive processes essential for their welfare [13]. To achieve this balance, it is necessary to understand the value of behaviours and cognitive processes to the welfare of captive animals. Veasey makes the case that if such a balance can be found, captive welfare will peak, potentially even exceeding that routinely experienced in the wild where life can frequently be “nasty, brutish and short” [13].

Tigers and other large carnivores, alongside cetaceans, primates and elephants, are often seen as emblematic of welfare compromises experienced by wild animals in captivity. This is likely in part because they are all iconic species which attract attention, but also because these species, and in particular large carnivores, have a propensity to express stereotypic behaviours which are widely perceived to be symptomatic of welfare compromises [34,35], including to the general public. In a review carried out across 20 species of terrestrial carnivores, Clubb and Mason estimated stereotypic pacing amongst captive Felidae to range between 10.5–48.0% of observed time and infant mortality to range between 20–42% in captivity [35]. Tigers paced for an average of 16.4% of observed time and experienced infant mortality rates of 32% [35]. Across these 20 species, they found infant mortality was best predicted by minimum home range size in the wild and the incidence of stereotypic pacing was best predicted by median daily travel distance in the wild, and came to the conclusion that a wide ranging lifestyle was a risk factor for poor welfare amongst carnivores in captivity [35]. Amur or Siberian tigers (*Panthera tigris altaica*) are the widest ranging Felidae and as such merit particular attention as their wide-ranging nature places them in double jeopardy in regard to conservation in the wild and welfare compromises in captivity. By way of illustration, despite living in a landscape with a far higher human population density, Bengal tigers (*Panthera tigris tigris*) currently outnumber Amur tigers by a factor of six to one [36,37] and inhabit home ranges that are frequently an order of magnitude smaller than their northern cousins’ [38,39]. Consequently, selecting species based on their suitability to captive environments is not without broader conservation implications.

Several studies have sought to examine the state of captive tiger welfare, but none thus far have provided clear and comprehensive guidance on how captive tiger welfare might be improved. Nonetheless, it has been demonstrated that indicators of impoverished captive tiger welfare were sensitive to habitat size [40,41], broadly supporting the conclusions of Clubb and Mason [35]. However, tiger welfare also appeared to be sensitive to habitat quality [40,42], as well as enrichment and feeding regimes [32,40,43,44,45,46,47,48], suggesting that much can be achieved to augment captive tiger welfare by understanding their psychological needs and manipulating their habitats and management accordingly [13,23,28]. In 2018, an assessment of the psychological priorities of Amur tigers was carried out at the Korkeasaari Zoo in Helsinki, Finland. The purpose of the Helsinki assessment was to better understand the needs of Amur tigers in captivity in order to inform the design of a new tiger habitat at the zoo, which might also establish new standards of best practice for the species. The Helsinki assessment also represented a unique opportunity to explore the challenges faced by wild carnivores and other wide-ranging species in captivity more generally, and to consider whether insights from this assessment might also be applicable to other wide-ranging species.

## 2. Materials and Methods

Existing conceptual frameworks for understanding welfare, such as the five domains and five freedoms models, together with a variety of welfare assessment tools, are in broad agreement in regard to identifying and assessing the physical needs of animals [49,50,51]; however, neither model, nor any welfare assessment tool the author is aware of, outline a clear process for identifying species-specific psychological priorities. The absence of such a tool has had a tangible impact on animal care [13] and to address this deficit, an Animal Welfare Priority Identification System (AWPIS©) was developed to identify the psychological priorities as a means to direct management and habitat design to optimise welfare, and to assess the capacity of management strategies and facilities to safeguard welfare [23,28]. The AWPIS© assessment process is intended to complement existing frameworks pertaining to physical wellbeing, whilst providing much needed clarity on the psychological priorities for species. Veasey argues that the output of this process allows, for the first time, physical and psychological priorities to be methodically optimised according to anticipated net welfare impacts [13,23,28].

AWPIS© is based upon the relationship between evolution, motivation and animal welfare, whereby behaviours or cognitive processes of high evolutionary significance will be highly and potentially frequently motivated for (see [23,52,53,54,55,56,57,58,59,60,61,62]). Consequently, if animals are frustrated in their desire to express behaviours or cognitive processes, the welfare impact of that frustration will be broadly proportional to its evolutionary significance [23].

This methodology utilises a Delphi-based review undertaken by a panel of experts (see [63]), who rank behaviours and cognitive processes from 1–5 according to guidelines tailored for each of the 12 assessment criteria. Collectively, these 12 criteria systematically assess the evolutionary significance, motivational characteristics and established welfare impacts of individual behaviours and cognitive processes (where they have been demonstrated for a species). Of these 12 criteria, seven consider insights from the species’ behavioural ecology in the wild, including survival impact, reproductive impact, risk of expression, energy expended, time expended, prevalence and frequency of expression of behaviours and cognitive processes amongst a free ranging population. Three criteria draw on insights from both captive and wild populations, including innateness of the behaviour or cognitive process, their motivational strength and motivational origins. The final two criteria consider insights gained from research undertaken on captive animals and consider the positive impacts of expression and the negative impacts of being prevented from expressing a behaviour or cognitive process. Where panellists are unable to assess a particular behaviour or cognitive process by a specific criterion, it is marked as unknown and their contribution to the final evaluation is removed. Utilising an algorithm which adjusts the weighting of each of the 12 assessment criteria, AWPIS© combines the data from the panel into a single value (see also [64]) for each behaviour or cognitive process, reflecting its psychological significance to the species.

Whilst metrics pertaining to health and behaviour are routinely used to assess welfare, in order to determine psychological priorities, it is also necessary to consider the underlying cognitive processes that generate behaviours. Cognitive processes are those mental processes which include learning, problem solving, empathy, discrimination, expectation, etc., and which have evolved to help organise behaviours so animals can deal with the external world in a flexible way by eliciting the most effective behavioural responses [65,66], and have been shown to be important to welfare [15,67]. Uniquely, AWPIS© assesses the relative value of both behaviours and cognitive processes to an individual’s welfare, with two of the 12 assessment criteria relating to time and energy expenditure being automatically eliminated from the assessment of purely cognitive processes.

The behaviours and cognitive processes utilised in the Helsinki assessment were selected by the author according to biologically meaningful categories likely to be relevant to any potential management applications. They were subsequently reviewed and agreed upon by the panel at the start of the assessment. It should be noted that the list generated is not an ethogram and is intended for an entirely different purpose. An ethogram is typically used to study the activity of individual animals and is comprised of a list of mutually exclusive behaviours [68]. In contrast, the categories utilised in an AWPIS© assessment are intended to identify psychological priorities, and as such, is not restricted to behaviours. Furthermore, because behaviours and cognitive processes may be expressed simultaneously but are still worthy of independent consideration, the categories are not necessarily mutually exclusive.

To illustrate the process of compiling categories of behaviour and cognitive processes, the process undertaken for categorising behaviours and cognitive processes associated with the acquisition of food for Amur tigers is described. For the purpose of the Helsinki assessment, these behaviours and cognitive processes were broken down into three stages: seeking food, securing food and consuming food. Each of these stages were further broken down into constituent behaviours and cognitive processes where appropriate. The first phase, for the purpose of this assessment, is referred to as foraging and was defined as the seeking of opportunities to acquire food. Foraging encompasses multiple behaviours and cognitive processes that can occur simultaneously but may also be expressed independently in different contexts; these include walking, olfaction, making decisions, gathering information, learning, etc. However, these behaviours and cognitive processes are considered as a collective in relation to foraging as the individual component behaviours and processes are rarely expressed in isolation while foraging and cluster in a biologically meaningful way that is likely to be reflected in any management consideration. However, because many of these component behaviours and cognitive processes can also occur in contexts outside of foraging, some are also considered independently in the assessment. As an example, olfaction may be utilised by tigers to find food or a mate, and so it and other such behaviours and cognitive processes also need to be considered independently. The next phase of feeding encompasses the acquisition of food, which for tigers, is collectively referred to here as hunting. By way of contrast, these constituent behaviours (stalking prey, chasing prey and killing prey) do not occur in any other context, and typically occur in a sequence; unlike the activities in foraging, they are mutually exclusive. As a result, they can be reviewed independently and aggregated as a collective category (hunting) for subsequent analysis. The final consummatory phase is comprised of carcass processing and eating. Whilst carcass processing and eating are, to some extent, contemporaneous in their expression, the feeding of processed chow to tigers illustrates that eating does not require processing, and because neither behaviour occurs in any other context, there is a value in evaluating the significance of each element separately. However, as with the other phases of feeding, these constituent behaviours are also aggregated for analysis under the eating category.

Cognitive processes exclusively linked to specific behaviours are not considered independently from those behaviours as they are effectively considered through them. For example, foraging will be a highly effective proxy for the cognitive state, covering the desire to find food, together with the variety of cognitive process that foraging would entail, because foraging will be the preferred manifestation of those processes. Other behaviours that were considered to be notable for having substantial cognitive components to them for Amur tigers were territorial maintenance and marking, stalking prey, play, mating and fighting. Some cognitive processes were considered in isolation as the manifestation of the cognitive process might trigger a variety of behaviours, which even if an associated behaviour is catered for, does not necessarily mean the associated cognitive process is, and these cognitive processes may be of value to the individuals’ welfare in their own right [15,67]. The categories that were designated as cognitive processes for Amur tigers were exploring, learning, choice/decision making, watching/observing, flehmen/smelling, problem solving and socialising.

Accordingly, each of the behaviours and cognitive processes assessed for Amur tigers were aggregated into four groups: those considered to be physiological necessities essential for survival in the wild and captivity, those considered to be predominantly cognitive in nature and for which there might be a diverse set of behavioural manifestations, those behaviours with a substantial cognitive component to them and those behaviours with a limited cognitive component to them that in of themselves are not essential for survival in the wild or captivity. Furthermore, since attempts to replicate aspects of hunting are so widespread in captive tiger enrichment [33], behaviours and cognitive processes associated with feeding were assessed individually and as groups to better determine where enrichment priorities should be focused to maximise welfare. The categories of the assessed behaviours and cognitive processes are summarised in Table 1.

Since a principle purpose of the Helsinki assessment was to understand the needs of Amur tigers in captivity, panellists were asked to consider the needs of Amur tigers as a single entity, including both sexes and all age classes, rather than considering specific cohorts. If an understanding of the needs of a particular sex or age class was required, the assessment would target the needs of that cohort specifically, and behaviours and cognitive process would have been categorised accordingly. However, where behaviours or cognitive processes were unique to a specific cohort, such as suckling and mating, panellists identified the cohort in the assessment, and so the importance of mating is only considered in relation to adult tigers, and suckling is only considered in relation to juvenile tigers. For categories such as play and learning which can occur throughout life but which have different emphases at different life stages, a lifetime species-based assessment such as this will reflect the needs of the species rather than a specific cohort, which needs to be considered in the interpretation of the output.

In total, 12 panellists participated in the two-day Helsinki AWPIS© assessment for Amur tigers. On the first day, an introduction to the assessment process was provided by the facilitator who also summarised current research pertaining to the welfare of tigers in captivity. Other speakers presented on tiger management at Korkeasaari Zoo and behaviour and ecology in the wild. Participants also toured the tiger facilities and learnt first-hand about tiger management at the Korkeasaari Zoo. The second day was dedicated entirely to the AWPIS© assessment for Amur tigers.

The panellists represented three zoos holding Amur tigers, one in situ non-governmental organisation working with Amur tigers in the Russian Far East (Wildcats Conservation Alliance), and one animal welfare non-governmental organisation (Wild Welfare). It is unfortunate that in situ expertise was not better represented, as previous assessments have shown that a predominance in ex situ expertise can potentially skew specific results, reflecting the experiences of the panellists rather than the needs of the species being assessed [23]. However, these skews typically relate to specific behaviours, and overall, there tends to be good agreement between in situ and ex situ experts across all categories as a whole [23]. Furthermore, it was hoped that the introductory day in which presentations were given on behavioural ecology and research in situ, together with a summary of the current literature relating to tiger welfare in captivity, would establish a more uniform, holistic and up-to-date understanding of the species for all participants.

## 3. Results

**Figure 1:**
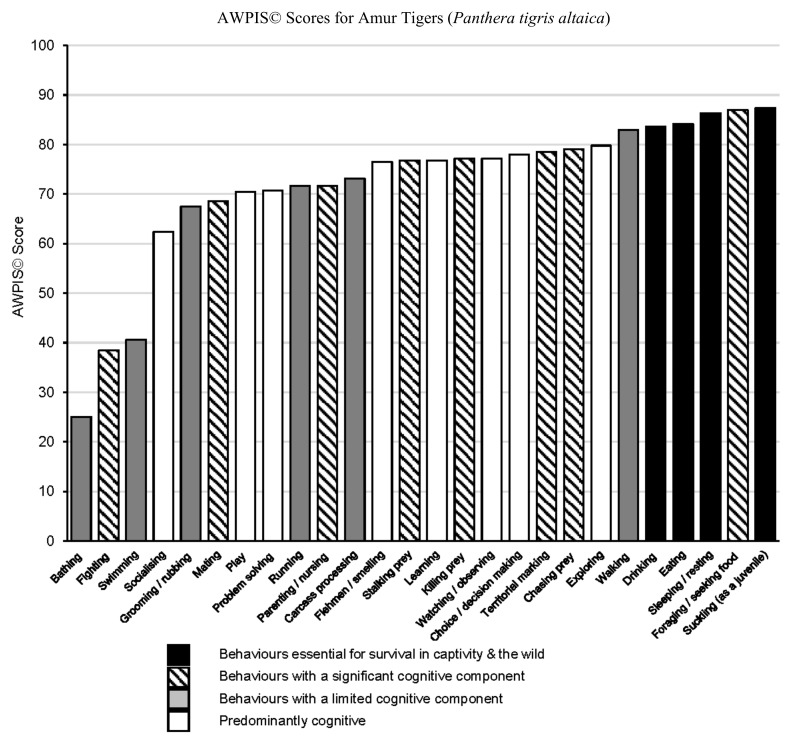
AWPIS© scores for Amur tigers.

Table 1 outlines the categories of behaviour and cognitive processes assessed, together with the consolidated AWPIS© scores derived from the Helsinki assessment of Amur tiger welfare priorities, which are also presented in graphical form in Figure 1.

Foraging emerges as the greatest psychological priority for adult tigers, followed by behaviours identified as being essential for survival (physiological necessities) in both captive and wild scenarios, closely followed by walking, exploring, chasing prey, territorial marking and choice/decision making.

**Figure 2:**
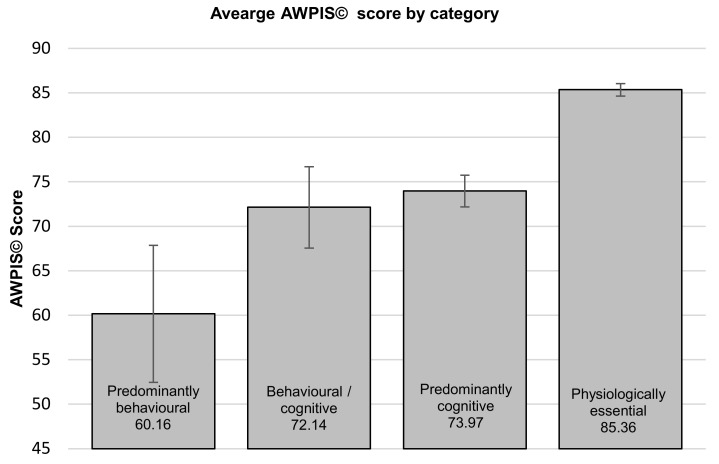
Comparison of group means ± standard error across the four categories of behavioural and cognitive priorities (ANOVA (F(3,22) = 2.747, *p* = 0.067).

**Figure 3:**
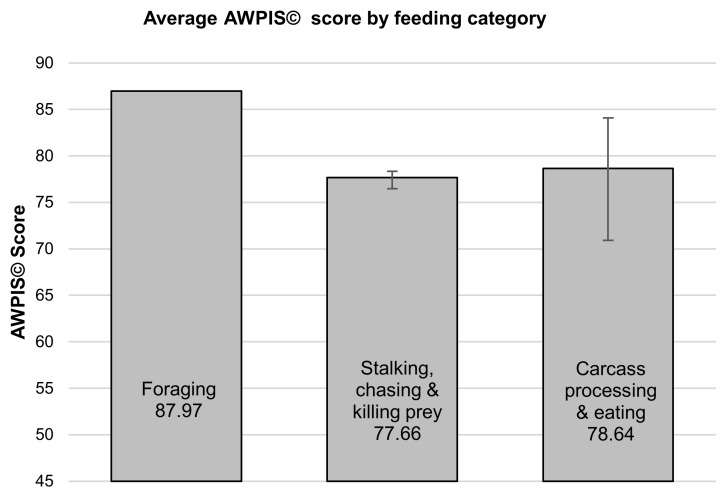
Comparison of group means ± standard error across three categories of priorities linked to the acquisition of food (ANOVA (F(2,3) = 1.617, *p* = 0.334).

There were no statistically significant differences between group mean AWPIS© scores across the four categories of priorities (see Figure 2) as determined by one-way ANOVA (F(3,22) = 2.747, *p* = 0.067), although physiological necessities appear to represent a greater psychological need than behaviours with a limited cognitive component to them. Neither was there a significant difference between the AWPIS© scores across the three categories of food acquisition behaviours and processes as determined by one-way ANOVA (F(2,3) = 1.617, *p* = 0.334) (see Figure 3).

**Figure 4:**
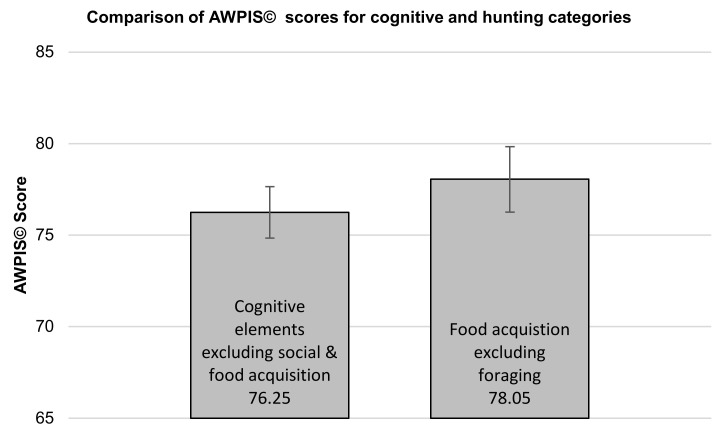
Comparison of group means ± standard error between cognitive processes and hunting-related behaviours, excluding social and foraging priorities (unpaired t-test; t(8) = −0.793, *p* = 0.451).

In view of the fact that felid enrichment is largely dominated by activities linked to the acquisition of food [33], it was considered important to assess the relative significance of priorities linked to the physical acquisition of food with those aspects of an Amur tiger’s life with a greater cognitive component to them, as this is a component that is frequently overlooked in terms of enrichment [67]. In order to do this, it was necessary to adjust overlapping categories; social behaviours were excluded from cognitive processes because being a relatively solitary species, their inclusion could artificially depress the significance of cognitive processes to Amur tigers, and foraging was excluded from both hunting and cognitive processes because it is arguably as much a cognitive process as it is a behavioural process linked to food acquisition. There was no significant difference between those aspects of a tiger’s life with substantial cognitive components to them, excluding categories linked to the acquisition of food (choice/decision making, learning, problem solving, territorial maintenance/marking and watching/observing; (mean = 76.25; standard error = 1.41)) and those behaviours linked to hunting and consuming food (eating, killing prey, stalking prey, chasing prey and carcass processing; (mean = 78.05; standard error = 1.79)) as determined using a non-paired t-test (t(8) = −0.793, *p* = 0.451; see Figure 4), suggesting that behaviours linked to the acquisition of food, and behaviours and processes with a significant cognitive component to them, are equally important to Amur tigers.

## 4. Discussion

When Tipu Sahib, the Sultan of Mysore (175–1799), stated that it was “better to live one day as a tiger than a thousand years as a sheep”, he had likely been referring to wild tigers free to roam vast forested habitats. Whether he would have made such a statement in regard to captive tigers it is impossible to know, but the evidence suggests that the life of a wild tiger will be markedly different from the life experienced by a captive one, and that those differences will likely have welfare consequences. The Helsinki assessment sought to understand where those differences might lie and how they might impact welfare in order to better understand and provision for the needs of Amur tigers in captivity, and potentially other wide-ranging species. Accordingly, the assessment identifies a number of priority considerations for captive Amur tiger welfare management.

Firstly, the four behaviours categorised as being essential for survival in both captivity and the wild for Amur tigers were identified as being amongst the top five priorities, suggesting that they possess a psychological dimension to them worthy of consideration in their own right. Whilst these behaviours are invariably catered for in captivity, sufficient to satisfy the animals’ physiological needs, there is no guarantee they are catered for sufficiently enough to fulfil the psychological/cognitive needs associated with those behaviours [67]. The high AWPIS© scores for these behaviours should alert those responsible for the care of captive Amur tigers to give greater consideration to the psychological and cognitive aspects of those behaviours over and above merely satisfying their physical consequences, and it would seem reasonable to conclude that this would most likely apply to the majority of higher vertebrates.

The results also revealed cognitive opportunities are likely to be more important than has previously been considered the case based on the prevailing emphasis of tiger and carnivore enrichment [33], and at least on a par with behaviours associated with food acquisition, which dominate carnivore enrichment [33].

Moreover, foraging, a behaviour intrinsically linked to the acquisition of food but considered to have a significant cognitive dimension to it, was considered the highest scoring priority for adult Amur tigers.

Before considering how these insights could be applied to reimaging the management of captive Amur tigers and potentially other wide ranging species, it is worth reviewing how this assessment (in conjunction with the wider literature) should inform our thinking in relation to the factors influencing Amur tiger welfare, and that of other wide ranging animals and terrestrial carnivores.

### 4.1. The Impact of a Wide-Ranging Lifestyle

In their review of natural behavioural biology as a risk factor in the welfare of captive carnivores, Clubb and Mason had anticipated a dependency upon hunting for survival as being the most significant risk factor for compromised welfare [35]. Based on the prevailing emphasis of carnivore enrichment at this time, this suspicion was likely shared by the majority of captive carnivore managers with 79% of enrichment studies for terrestrial carnivores relating to food or artificial prey [33]. However, Clubb and Mason’s review of 20 species of terrestrial carnivores instead demonstrated that a wide-ranging, far-travelling lifestyle was a greater risk factor for compromised welfare than hunting ecology, influencing both infant mortality rates and time spent pacing in captivity [35]. The impacts of a far-ranging lifestyle are not an area of consideration recognisably covered by any carnivore enrichment identified in Shyne’s review [33]. Clubb and Mason’s conclusions are supported by the Helsinki assessment with the emergence of foraging as the most significant psychological priority for adult Amur tigers, with walking, exploring and territorial maintenance also ranking highly.

The relationship between pacing and the restriction of ranging opportunities amongst terrestrial carnivores in captivity has also been demonstrated within tigers as a species, with individuals held in smaller captive habitats tending to pace more than those in larger habitats [40,41]. However, whilst collectively the work of Clubb and Mason, Veasey and Breton and Barrot provide useful insights into potential welfare impacts associated with space restriction for tigers and carnivores generally, they are of limited use in tailoring management to improve captive welfare for distinct species and subspecies, over and above simply calling for more space [34,35,40,41]. This is perhaps especially true for tigers, as none of these studies distinguished between the various subspecies of tiger in their analyses despite the wide variation in natural ranging behaviour between them.

Amongst the terrestrial carnivores, Amur tigers are one of the widest ranging, in some cases covering an area exceeding 2500 km^2^ [69], likely only routinely exceeded by polar bears (*Ursus arctos*). By way of comparison, Clubb and Mason’s analyses used a generic figure for the home range size for tigers of just 48 km^2^ [35], most likely using data derived from multiple tiger subspecies including less wide ranging tropical subspecies such as the Bengal tiger, which may range as little as 16 km^2^ [38]. Since an animal’s home range is in effect a spatial representation of the behaviours required by it to maximise its fitness in the wild, it is reasonable to conclude that such a divergence in ranging behaviour amongst wild tigers requires us to consider the needs of each tiger subspecies differently. Therefore, Amur tigers have likely been done a disservice in aggregating them with other subspecies as the challenges they might experience in captivity are likely more acute than their tropical, less wide-ranging relatives under the same conditions.

The divergence in ranging behaviours amongst wild tigers will ultimately be a consequence of the differing prey density in the habitats that the subspecies reside (see [70,71]), which will in turn be influenced by latitude and productivity [41,71,72,73]. The extent to which this divergence in ranging behaviour is reflected in genetic adaptations of each subspecies to their respective environments as opposed to flexible, behavioural adjustments is difficult to determine; however, there are marked latitudinal differences in size, appearance and genotype between the subspecies, with Sumatran tigers (*Panthera tigris sumatrae*) weighing around half as much as Amur tigers [74,75], for example. It would appear highly likely that differences in these heritable characteristics between the subspecies are not limited to their phenotype and are likely to include distinct heritable behavioural and cognitive adaptations that will translate into differential welfare implications between the subspecies in captivity. It is for this reason that as a minimum, the needs of tropical tiger subspecies need considering separately from the needs of the Amur tiger, which, because of their markedly wider ranging behaviour in the wild, merits particular attention.

Whilst the link between spatial restriction and indicators of impoverished welfare has been established for terrestrial carnivores and within the tiger species, it is important to consider whether it is the restriction in space per se that is the causal factor in compromising the welfare, or rather the consequential impact of reductions in habitat size on habitat quality.

Evidence from multiple sources appear to show that the habitat quality of captive carnivores can be augmented without increasing captive habitat size [33]. Within the constraints of the existing captive habitats, opportunities to investigate feeding devices throughout the day significantly reduced the incidence of pacing amongst captive tigers [47], as did bungee-carcass feeding [48], olfactory enrichment [44] and the provision of live fish and bones [32]. Furthermore, it has been shown that both increased habitat complexity and quality appeared to diminish pacing across a variety of tiger subspecies [40].

When considering the issue of enclosure size, it is also worth acknowledging that the physical constraints of captive habitats do not appear to limit locomotion in carnivores, particularly if pacing is included. Captive mink, for example, can pace more than four times the distance typically travelled in the wild [76], and captive tigers routinely cover greater distances in zoo enclosures than they would routinely walk in the wild. Amongst a mixed population of 38 captive tigers, individuals were recorded travelling up to 19 km in just six hours of observation [41], far in excess of the figure for wild daily travel distance used by Clubb and Mason of 8.07 km [35] and the figure recorded for wild Amur tigers who averaged 13.5 km during the course of a 24 h period in optimal conditions [39].

The Helsinki assessment clearly identifies foraging and walking as welfare priorities for Amur tiger care in captivity. For Amur tigers, foraging requires ‘walking’, but as defined in the assessment, it is distinct from it when walking does not involve searching for food. Therefore, for the purpose of this assessment, foraging includes walking when it is combined with significant cognitive and goal directed components linked to the acquisition of food such as navigation, decision making, information gathering, seeking, sensing etc., that walking alone need not require.

Foraging and walking are amongst the most obvious candidate behaviours to fall victim to a compression in ranging opportunities associated with captivity amongst tigers, but the results from the Helsinki assessment suggest that a reduction in foraging opportunities is likely to have the greatest impact on tiger welfare. From a biomechanical perspective, pacing and walking are directly comparable, and perhaps reassuringly, the physical constraints of most captive environments do not appear to prevent tigers covering distances comparable to those undertaken by their wild relatives. However, evidently, pacing in captivity and walking and foraging in the wild are not comparable from a cognitive perspective, and it is unlikely that even the most generous and enriched of existing captive tiger habitats will satisfy the cognitive processes routinely associated with travel in the wild.

Thus its proposed here that the apparent impact of habitat size on pacing in tigers is not a consequence of habitat size per se, but rather the reduction in cognitive opportunities that likely covary with size, based on the evidence that captive habitat size does not limit locomotion, and indicators of poor welfare in captivity can be reduced by augmenting habitat quality. This may appear to be an academic distinction, but it has important consequences in terms of management and habitat design; whilst the compression of home range size appears to be a risk factor for tiger welfare, counterintuitively, management and design solutions to mitigate the impact of captivity on tiger welfare are unlikely to be optimised simply by increasing habitat size within the constraints of most zoos. Instead, the greatest welfare gains are likely to be achieved by ensuring the behavioural, psychological and cognitive opportunities routinely experienced and motivated for in the wild, and as identified in the Helsinki assessment, are adequately catered for in captivity.

### 4.2. Hunting, Feeding and Foraging

Behaviours linked to the acquisition of food are already the principle focus of most enrichment programs for carnivores [33] and for which beneficial effects for tiger welfare have been reported [32,33,46,48]. However, for wild tigers, who are largely ambush predators [30], the process of securing food is underpinned by a diverse array of behaviours and cognitive processes beyond simply killing prey. Most obviously, these will include appetitive behaviours associated with hunting, including finding and selecting prey (or carcasses to steal or scavenge), stalking/waiting for prey, a typically short chase followed by killing the prey, carcass processing and potentially carcass concealment and defence behaviours. Beyond these behaviours and associated cognitive processes, the tiger must also maintain a real-time awareness of prey distribution and density, establish and maintain a territory of sufficient size to sustain the tiger (and, where necessary, dependant cubs), gather information relating to both competitors and food sources, navigate within that territory, predict outcomes and make decisions.

Thus, whilst it may seem surprising that in the Helsinki assessment that hunting related behaviours (chasing, killing and stalking prey) were ranked as lower psychological priorities than foraging, exploring, territorial maintenance/marking and decision making, these rankings are reflective of the greater time and energy expended expressing these behaviours and their associated cognitive processes, as well as their arguably greater evolutionary impacts; without interfacing with their environment in a thinking, dynamic manner, tigers would not encounter opportunities to hunt sufficiently to ensure survival, nor access mates frequently enough to maximise reproductive output. In contrast, wild tigers will readily scavenge if the opportunity is presented to them (see [37,77,78]), and so it is conceivable that tigers might survive without stalking, hunting or killing prey for a period of time by relying on carrion or by usurping other predators from their prey. Whilst hunting will evidently be important to tigers, there is likely more flexibility in regard to a captive tigers’ need to express hunting behaviours, that is to say chasing and killing prey, in comparison to their need to forage or interface with their environment in a dynamic thinking manner, a hypothesis supported by the Helsinki assessment. Furthermore, the motivation to hunt is likely sated instantaneously by the extrinsic stimulus of a meal, whereas the motivation to forage, explore and defend a territory is likely dependent upon more multifaceted intrinsic and extrinsic factors, ranging from stomach distension through to duration since last expression and other multisensory external cues that will likely persist in captivity and cannot be so easily manipulated. Nonetheless, hunting related behaviours still scored relatively highly in the assessment, and are likely to be inherently rewarding to express; as such, they could help provide captive Amur tigers with positive experiences, an essential component of good welfare (see [20,23,32,50,51]).

### 4.3. Cognitive Opportunities

The results from the Helsinki assessment demonstrated that cognitive priorities are, broadly speaking, equally important as behaviours associated with the acquisition of food. Given the ultimate dependency of food acquisition on both hunting behaviours and a wide array of cognitive processes, this is perhaps unsurprising. However, the Helsinki assessment indicates that foraging and other cognitive process are likely of greater importance to captive tiger welfare than might have been previously acknowledged based on enrichment typically used for captive carnivores [33,67]. Shyne’s review [33] appears to indicate that the majority of ‘behavioural enrichment’ programmes for carnivores fail to recognise the possibility that the cognitive processes associated with feeding in the wild may be of greater significance than the behaviours themselves. Whilst tiger enrichment devices such as feeding poles, bungee-carcass feeding and rope pulls may provide some beneficial behavioural enrichment linked to hunting behaviours [33,46,48], their cognitive benefits may be limited [67]. The work of Plowman and Knowles [79], Jenny and Schmid [47] and Damasceno et al. [44] have however shown that attempting to replicate cognitive aspects of feeding and foraging through olfactory enrichment and scattered feeding devices can be beneficial to tiger welfare.

### 4.4. Hydrophily

The Helsinki assessment appears to highlight a potential discrepancy between the likely importance of bathing and swimming to Amur tigers and the prevailing narrative that captive tigers enjoy swimming or immersing themselves in water [42,80,81]. This apparent discrepancy could result from an inaccurate but widely held belief based on anecdote, an increase in water usage as a consequence of some aspect of the captive environment, or a failing in the assessment process itself. In regards to the latter, it is worth noting that pools are typically a requirement of tiger husbandry guidelines [80,81] and that this assessment was dominated by zoo professionals with experience managing Amur tigers in habitats in which pools are ubiquitous. As such, it seems unlikely that the results from the assessment would understate the importance of bathing and swimming were those pools being widely used.

Biolatti et al.’s claim that “the presence of a water pool with clean water was significantly associated with enhanced welfare” for captive tigers [42] appears to contradict the findings of the Helsinki assessment. However, this claim must in part be attributable to the fact they defined immersion in water as an indicator of enhanced welfare. They also claimed that even in habitats in which tigers did not use pools, that the “presence of a water pool with clean water encourages tigers to perform behaviours considered as indicators of enhanced welfare” [42]. However, in their review of potential causal factors impacting welfare measures, Biolatti et al. evaluated the habitat on a binary basis, considering only the presence or absence of a pool, shade, a platform and a log, and whether or not the habitat was bigger than 1000 m^2^ [42]. It is likely that such a reductive approach to investigating the impacts of environmental quality on welfare will lead to interpretive challenges, and it is perhaps more likely that habitats with pools containing clean water also benefitted from other features not identified in their binary analysis of environmental quality that might have a positive impact on welfare. This could include opportunities to be out of sight and interact with a potentially more complex environment. The low incidence of pool use (1.7% across the population of tigers studied) casts further doubt over the assertion that access to a pool is the most significant driver or captive tiger welfare [42].

The Helsinki assessment is not the first to suggest that the importance of water usage (excluding drinking) may have been overestimated for terrestrial mammals by prevailing captive management priorities [23,28]. Pools are popular in zoo habitats because they create a sense of aesthetic completeness and naturalness which is known to be important to public perceptions of habitat quality [1,2]; water is essential for survival after all, and pools also represent tangible evidence to an increasingly discerning public of an apparent investment in welfare. However, despite the analysis of Biolatti et al. [42], evidence supporting the welfare benefit for tigers beyond a potential thermoregulatory role is absent or weak, particularly when considering the harsh climate Amur tigers naturally live in would logically limit their use of open water. Veasey points out that a potential increase in water use in captivity might be a consequence of captive animals encountering water more frequently, having more time to make use of water when not constrained by the demands of survival in the wild, being exposed to less risk when using water and having greater need to interact with water to thermoregulate in habitats that frequently have less canopy cover than they might have had access to in the wild [23]. It is also possible that captive animals may utilise the pools as alternative outlets for other potentially frustrated motivations, and bathing or swimming may or may not represent perfectly acceptable alternative outlets. Any evidence of hydrophily in captive tigers, if indeed it exists, may actually represent the expression of ‘luxury behaviours’ and a potential positive welfare outcome of the captive state [23].

However, the relatively low scoring of bathing and swimming for Amur tigers from the Helsinki assessment and the low pool use identified by Biolatti et al. [42] should not necessarily lead to the elimination of pools, particularly as the choice they afford tigers likely has an inherent welfare value [82,83,84], pools provide opportunities for enrichment [32] and they may also have important thermoregulatory roles not required in the wild. Rather, the Helsinki assessment should result in a greater understanding of the potential net welfare value of bathing and swimming to captive Amur tigers, and resources should be prioritised accordingly. Thus, with finite resources available (and depending upon climate), investing in foraging opportunities for Amur tigers is likely to yield greater welfare returns than filtered pools with underwater viewing, for example. This outcome should also serve to caution against the use of evidence founded on anecdote in determining management strategies and best practice guidelines.

### 4.5. Application to Habitat Design and Management

The principle intent of the Helsinki assessment was to provide a framework in which a new habitat could be designed for the zoo’s Amur tigers that pushed the limits of existing conceptions of best practice. Here, a vision is described of how Amur tiger management might be conceivably be recalibrated in captive environments to address the key priorities revealed by the assessment, and in particular, by providing appropriate foraging and cognitive opportunities as well as behaviours and cognitive processes linked to hunting.

The physical constraints of captive environments are evidently central to the consequential welfare challenges tigers (and indeed most species) face in captivity, either directly, or more likely indirectly as a consequence of an associated reduction in opportunities relevant to welfare. The question, therefore, is whether it is possible to address the lack of space, or species appropriate complexity that covaries with reductions in space, whilst remaining within the constraints of what is realistically achievable within a captive environment? Here an approach is outlined that has the potential to do exactly that; through an entirely novel methodology, the effective habitat size (as opposed to the actual habitat size) is increased within the constraints of a generously proportioned captive habitat.

Tigers range widely over territories to follow prey populations, find mates and to defend these finite resources from other tigers. This ranging behaviour will encompass a wide range of behaviours and cognitive processes identified as priorities to greater or lesser degrees during the course of the Helsinki assessment and, most notably include foraging, walking, exploring, territorial maintenance/marking, making decisions, watching/learning, olfaction and socialising. The habitats in which Amur tigers range are frequently highly vegetated and covered in deep snow, requiring tigers to follow well established ‘game-trails’, a behaviour long utilised by field scientists in the positioning of camera traps, and poachers in the positional of snares [85].

It is proposed here that within the footprint of a large captive habitat, a network of pathways could be established bounded by barriers concealed with, or comprised of, densely planted vegetation such as bamboo. This network of paths would form the equivalent of a maze, however, at certain key points along this network of paths, electronic gates linked to motion sensors would be positioned, allowing the route through the space to be adjusted to create a potentially infinitely variable, programmable route.

At key points, these gates would provide tigers with opportunities to make choices over direction of travel, with different outcomes at different distances from the points of choice, including further encounters with gates revealing a cascading series of opportunities for choice. This would attempt to replicate, to a previously unparalleled degree, the complexity of a wild environment, generating the opportunity for captive tigers to make choices, learn, navigate, explore and to experience a sense of purpose and achievement. Using such a model, tigers could choose to undertake journeys to access resources such as platforms, denning areas, shade, ponds and feeding opportunities configured to replicate hunting contingencies [46] with, for example, variable reinforcement schedules. It is the author’s personal opinion that in seeking to improve the welfare of one species (the tiger), it should not occur to the detriment of other individuals, and as a result, the live feeding of prey (including fish) is not acceptable in attempting to replicate hunting contingencies and opportunities. Furthermore, effort should be made to source food for tigers (and all other zoo animals) from suppliers capable of guaranteeing the appropriate welfare of livestock used.

To further increase the sense of place and increase the cognitive opportunities for tigers, a variety of olfactory stimuli could be utilised at predetermined points along the journey selected by the tiger to assist with it developing a multisensory mental map of its habitat. Thus, a 2 km journey towards a platform might be linked to a specific scent, whereas a 4 km journey to a water source might be linked to another biologically appropriate scent released from programable units within the habitat. At key anchor points along the route, ‘scratching posts’ would be available for the tigers to satisfy territorial maintenance behaviours and cognitive processes, identified as being of value to captive Amur tiger welfare in the Helsinki assessment.

Collectively, this dynamic, multisensory space could be configured to ensure captive tigers would be provided with both reason and opportunity to make journeys comparable to the daily ranging behaviour of wild tigers, and would re-establish the contingency between cognitive processes and behaviours and goal directed outcomes, a feature lacking in most zoo habitats and widely believed to be relevant to captive animal welfare (see [67,86]). In creating a space in which spatial complexity is dynamic and programmable, the effective space for the animal will be substantially larger than the actual space available to it. Such a design, in which animals are provided the opportunities to solve their own challenges as opposed to being presented with solutions by carers, would be the first serious attempt to address the longstanding challenge of the impact of reduced space on captive tiger welfare through the application of applied ethology. Furthermore, since Amur tigers experience challenges shared by many other wide-ranging species, it is anticipated that such an approach, if proven to be successful, could have wider applications.

## 5. Conclusions

Evidence suggests wide ranging species such as Amur tigers face welfare challenges as a result of habitat compression in zoos. Whilst its yet not clear whether zoos will ultimately be able to fully satisfy the psychological needs of species subject to substantial habitat compression, the AWPIS© based review undertaken here does provide insights into how those challenges might be addressed. For Amur tigers, the review makes the case that the prevailing management systems have failed to adequately address important behaviours and cognitive processes linked to foraging and territory maintenance, and that more attention should be paid to cognitive processes generally. The review also suggests that within the constraints of a typical zoo, the greatest improvements in captive Amur tiger welfare are likely to be achieved in addressing habitat quality in a taxa-specific manner, rather than marginally increasing the amount of space available to tigers. The review subsequently proposes novel solutions to long term and well documented welfare challenges for what is ultimately one of the most iconic species routinely held in zoos, that warrants further exploration.

Historically, progress in the provisioning of captive animal welfare has been a gradual process based on iterative adjustments to existing management systems. In contrast, as demonstrated here, the AWPIS© tool helps identify strategies to improve animal welfare which are instead centred around the fundamental needs of the species as a product of their evolutionary history. Furthermore, by evaluating the extent to which animals are able to experience the psychological priorities identified using AWPIS©, the methodology also offers up a unique welfare assessment tool. The AWPIS© methodology could therefore dramatically accelerate improvements in animal welfare attainment, as well as enhancing animal welfare assessment in a manner that better reflects the behavioural ecology of each species being considered.

## Figures and Tables

**Table 1:** Animal Welfare Priority Identification System (AWPIS©) rankings for Amur tigers.

Behaviour/Cognitive Process	Definition (Where Required)	Category	AWPIS Score
Suckling (juveniles only)	The process of a juvenile acquiring milk, typically from its mother	Physiological need	87.39
Foraging	Actively seeking opportunities to acquire food whether by hunting, scavenging or stealing	Behavioural/cognitive	86.97
Sleeping/resting	-	Physiological need	86.33
Eating	-	Physiological need	84.11
Drinking	-	Physiological need	83.61
Walking	-	Behavioural	83.00
Exploring	Traveling through an unfamiliar area and gathering information	Cognitive	79.78
Chasing prey	-	Behavioural/cognitive	79.00
Territorial maintenance/marking	Patrolling a territory, monitoring the location of conspecifics, marking the animal’s presence in its habitat	Behavioural/cognitive	78.55
Choice/decision making	Selecting between options	Cognitive	77.95
Watching/observing	-	Cognitive	77.20
Killing prey	-	Behavioural/cognitive	77.19
Learning	The acquisition of knowledge or skills through observation, experience, or being taught	Cognitive	76.81
Stalking prey	-	Behavioural/cognitive	76.80
Flehmen/smelling	Olfaction not linked to foraging, including assessing the status of conspecifics	Cognitive	76.46
Carcass processing	Processing food items prior to ingestion	Behavioural	73.17
Parenting/nursing	Active care of juveniles including feeding	Behavioural/cognitive	71.66
Running	-	Behavioural	71.61
Problem solving	Identifying solutions to complex challenges	Cognitive	70.73
Play	-	Cognitive	70.39
Mating (adults only)	-	Behavioural/cognitive	68.51
Grooming	-	Behavioural	67.51
Socialising	Participation in any social activity with conspecifics other than in the course of mating and parenting	Cognitive	62.42
Swimming	Immersing in water to travel	Behavioural	40.61
Fighting	Aggressive interaction between conspecifics, excluding play	Behavioural/cognitive	38.45
Bathing	Immersing in water for any other purpose than acquiring food or swimming	Behavioural	25.07
